# Supplementary figures and images for: Large language model detects previously undiagnosed heart failure with preserved ejection fraction in patients with metabolic-associated fatty liver disease: A multicenter cohort study
Source: PLOS Digit Health. 2026 Mar 31;5(3):e0001317. doi: 10.1371/journal.pdig.0001317 (PMC13037960; doi:10.1371/journal.pdig.0001317)

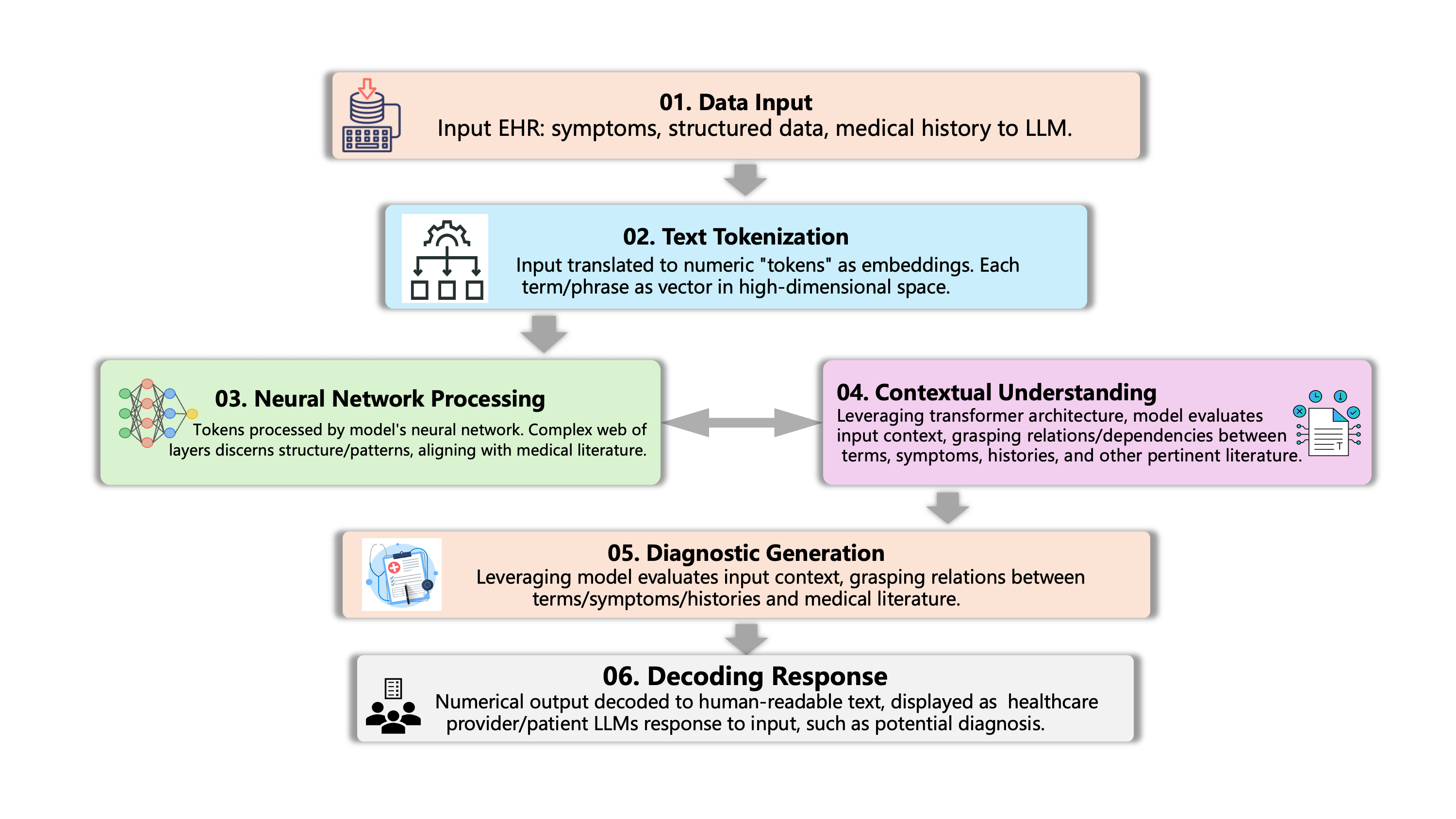

Supplement: S1 Fig — The figure illustrates the inference pipeline used in this study. Structured and unstructured electronic health record (EHR) data were provided as input to MedGuide-14B, which processed the information through tokenization, transformer-based contextual representation, and probabilistic inference. The model output a probability score representing the likelihood of heart failure with preserved ejection fraction (HFpEF). This schematic reflects the model invocation and inference process only and does not depict model training or fine-tuning procedures. (TIFF) [file pdig.0001317.s001.tiff]
