# Supplementary material for: Large language model detects previously undiagnosed heart failure with preserved ejection fraction in patients with metabolic-associated fatty liver disease: A multicenter cohort study
Source: PLOS Digit Health. 2026 Mar 31;5(3):e0001317. doi: 10.1371/journal.pdig.0001317 (PMC13037960; doi:10.1371/journal.pdig.0001317)
Supplement: S1 File — This document provides additional methodological information not fully described in the main Methods section, including details on data preprocessing and normalization, handling of missing data, MedGuide-14B model invocation and inference workflow, standardized prompt structure, probability threshold selection for HFpEF classification, and supplementary statistical analyses. (DOCX) [file pdig.0001317.s002.docx]

**S1 Method**

**Model Invocation and Inference Workflow**

MedGuide-14B was invoked using a standardized inference workflow that integrates both structured and unstructured electronic health record (EHR) information. Structured inputs included demographic variables, comorbidities, laboratory measurements, and echocardiographic parameters, while unstructured inputs comprised free-text clinical narratives such as presenting symptoms, physician assessments, and imaging report descriptions.

For each patient, the model generated a continuous probability score ranging from 0 to 1, representing the likelihood that the patient met the European Society of Cardiology (ESC) diagnostic criteria for heart failure with preserved ejection fraction (HFpEF). A fixed probability threshold of 0.70 was used to classify patients as HFpEF-positive. This threshold was selected *a priori* to balance sensitivity and positive predictive value based on internal validation analyses and was applied consistently across all evaluation cohorts. No post hoc threshold optimization or cohort-specific tuning was performed.

The model outputs were used solely for classification and risk stratification purposes. All analyses were conducted using de-identified, group-level data, and no patient-level identifiers were included at any stage of model inference or evaluation.

A schematic overview of this inference workflow is provided in Supplementary S1 Fig.
