# Supplementary material for: Large language model detects previously undiagnosed heart failure with preserved ejection fraction in patients with metabolic-associated fatty liver disease: A multicenter cohort study
Source: PLOS Digit Health. 2026 Mar 31;5(3):e0001317. doi: 10.1371/journal.pdig.0001317 (PMC13037960; doi:10.1371/journal.pdig.0001317)
Supplement: S2 File — This document describes the blinded adjudication workflow and consensus procedures used for independent review of MedGuide-identified HFpEF cases, including reviewer selection criteria, case assignment, and discordance resolution. (DOCX) [file pdig.0001317.s003.docx]

**S2 Method**

**Clinical Validation and Adjudication Workflow**

To evaluate the clinical validity of model-identified HFpEF cases, a randomly selected subset of 500 patients classified as HFpEF-positive by MedGuide-14B underwent blinded clinical adjudication. Board-certified cardiologists independently reviewed de-identified clinical summaries without access to model predictions or probability scores.

Adjudicators determined HFpEF status based on ESC diagnostic criteria, classifying each case as confirmed or not confirmed HFpEF. Discrepancies between reviewers were resolved through consensus discussion. Validation outcomes were summarized using group-level statistics, and no individual patient data were disclosed.
