# Supplementary material for: Large language model detects previously undiagnosed heart failure with preserved ejection fraction in patients with metabolic-associated fatty liver disease: A multicenter cohort study
Source: PLOS Digit Health. 2026 Mar 31;5(3):e0001317. doi: 10.1371/journal.pdig.0001317 (PMC13037960; doi:10.1371/journal.pdig.0001317)
Supplement: S3 File — This document presents detailed calibration procedures and bin-level assessment of MedGuide-14B predicted probabilities, along with representative anonymized patient-level examples illustrating the full EHR input, model inference process, handling of missing diagnostic elements, and final adjudicated interpretation. (DOCX) [file pdig.0001317.s004.docx]

**S3 Method**

**Calibration Analysis Details**

Model calibration was assessed to evaluate the agreement between predicted probabilities and observed outcomes. Calibration performance was quantified using the Brier score and a decile-based calibration approach. Predicted probabilities were partitioned into ten equally sized bins according to their predicted risk, and within each bin, the mean predicted probability was compared with the observed HFpEF prevalence.

Calibration analyses were performed using de-identified summary statistics and are reported at the group level. The numerical values underlying the calibration assessment, including bin-level predicted and observed proportions, are publicly available in the accompanying data repository.
