# Supplementary material for: Large language model detects previously undiagnosed heart failure with preserved ejection fraction in patients with metabolic-associated fatty liver disease: A multicenter cohort study
Source: PLOS Digit Health. 2026 Mar 31;5(3):e0001317. doi: 10.1371/journal.pdig.0001317 (PMC13037960; doi:10.1371/journal.pdig.0001317)
Supplement: S4 File — This document provides representative anonymized patient-level examples illustrating the model’s diagnostic reasoning under conditions of complete and partially missing clinical information, demonstrating how MedGuide-14B handles uncertainty in real-world EHR data. (DOCX) [file pdig.0001317.s005.docx]

**S4 Method**

**Anonymized Patient-Level EHR Inputs and MediGuide-14B Outputs**

To illustrate how MediGuide-14B operates on real-world, non-cardiology-centered electronic health records (EHRs), we provide two representative anonymized patient-level examples. These cases demonstrate the complete analytical pipeline, including raw EHR inputs, model reasoning and outputs, handling of missing diagnostic elements, and final adjudicated interpretation.

**Case A** represents a clinically adjudicated HFpEF patient with complete diagnostic evidence, while **Case B** illustrates a previously undiagnosed patient flagged by MediGuide-14B under partially missing data conditions.

**Case A: Clinically Diagnosed HFpEF (Gold-Standard Reference Case)**

**Patient ID:** P-220803-001
**Sex/Age:** Female, 66 years
**BMI:** 29.3 kg/m²
**Admission Date / Department:** 2024-08-12, Gastroenterology

**Admission Context and Raw EHR Inputs**

The patient was admitted due to repeatedly elevated alanine aminotransferase (ALT) and gamma-glutamyl transferase (γ-GT) detected during routine physical examinations over three months, accompanied by upper abdominal fullness and discomfort.
Over the preceding month, she reported exertional dyspnea after climbing one to two flights of stairs, need for an elevated pillow position during sleep, intermittent palpitations, and mild bilateral ankle edema resolving spontaneously in the morning.

**Past Medical History:**

Hypertension (10 years), treated with amlodipine 5 mg/day

Type 2 diabetes mellitus (5 years), treated with metformin

No smoking history; occasional alcohol consumption

**Vital Signs on Admission:**

Blood pressure: 158/92 mmHg

Heart rate: 78 beats/min

**Key Laboratory Findings:**

NT-proBNP: 968 pg/mL

ALT: 43 IU/L; γ-GT: 66 IU/L

HbA1c: 7.4%

Renal function: normal

**Imaging and Cardiac Evaluation:**

Echocardiography: LVEF 58%, LVMI 116 g/m², left atrial diameter 44 mm, E/e′ 17.8, SPAP 40 mmHg

Abdominal ultrasound: diffuse fatty liver

Chest X-ray: heart size at upper limit of normal

**Model Prompt (Task Definition)**

The model was instructed to analyze structured and narrative EHR data to assess the likelihood of heart failure with preserved ejection fraction (HFpEF) according to ESC 2016 diagnostic criteria, HFA-PEFF, and H2FPEF scoring systems. If complete scoring elements were unavailable, the model was permitted to classify cases as “suspected HFpEF” with explicit identification of missing items.

**MediGuide-14B Output Summary**

**ESC 2016 Criteria:** All three criteria fulfilled (symptoms/signs, LVEF ≥50%, objective evidence).

**HFA-PEFF Score:** 9 points (functional, morphological, and biomarker domains), confirming HFpEF.

**H2FPEF Score:** 4 points, indicating intermediate-to-high probability.

**Key Supporting Evidence Identified by the Model:**

Elevated NT-proBNP

Diastolic dysfunction (E/e′ >14)

Left atrial enlargement and left ventricular hypertrophy

**Missing or Unavailable Data**

Exercise stress testing

Polysomnography results

Thyroid and iron metabolism indices

**Final Adjudicated Interpretation**

The patient fulfills established ESC and HFA-PEFF criteria for HFpEF. The model’s output was concordant with the final clinical diagnosis.

**Case B: Previously Undiagnosed HFpEF Flagged by MediGuide-14B**

**Patient ID:** P-230107-014
**Sex/Age:** Male, 59 years
**BMI:** 28.8 kg/m²
**Admission Date / Department:** 2023-01-07, Gastroenterology

**Admission Context and Raw EHR Inputs**

The patient was admitted for evaluation of mild transaminase abnormalities and coarse liver echotexture detected during routine examinations. Over the preceding two months, he experienced mild exertional dyspnea after walking approximately 500 meters and reduced exercise tolerance.

**Past Medical History:**

Hyperlipidemia (on atorvastatin)

Obstructive sleep apnea (OSA), suboptimal CPAP compliance

No history of hypertension or diabetes

**Vital Signs on Admission:**

Blood pressure: 128/90 mmHg

Heart rate: 76 beats/min

**Key Laboratory Findings:**

NT-proBNP: 486 pg/mL

Mild ALT/AST/γ-GT elevation

Renal function: normal

**Imaging and Cardiac Evaluation:**

Echocardiography: LVEF 54%, LVMI 109 g/m², left atrial diameter 38 mm, E/e′ 16.2, SPAP 35 mmHg

Abdominal ultrasound: fatty liver

Chest X-ray: normal

**Model Prompt (Task Definition)**

Identical to Case A.

**MediGuide-14B Output Summary**

**ESC 2016 Criteria:** Symptoms, preserved LVEF, and objective evidence present → classified as suspected HFpEF.

**HFA-PEFF Score:** 4 points, suggesting intermediate probability requiring further evaluation.

**H2FPEF Score:** 1 point, reflecting low probability in isolation but discordant with biomarker and diastolic findings.

**Missing or Unavailable Data**

Exercise stress testing

Thyroid and iron metabolism indices

Objective assessment of CPAP adherence and response

**Final Adjudicated Interpretation**

The patient was classified as **suspected HFpEF**, with the model explicitly identifying incomplete diagnostic elements and recommending targeted follow-up testing. This case illustrates MediGuide-14B’s ability to flag clinically unrecognized HFpEF risk in non-cardiology settings under partially missing data conditions.
