# Supplementary material for: Large language model detects previously undiagnosed heart failure with preserved ejection fraction in patients with metabolic-associated fatty liver disease: A multicenter cohort study
Source: PLOS Digit Health. 2026 Mar 31;5(3):e0001317. doi: 10.1371/journal.pdig.0001317 (PMC13037960; doi:10.1371/journal.pdig.0001317)
Supplement: Attachment — Submitted filename: PDIG-D-25-00681_reviewer comments.pdf [file pdig.0001317.s006.pdf]

Review Comments to the Author

Thank you for submitting this well-prepared and clinically relevant manuscript, *"Large language model detects previously undiagnosed heart failure with preserved ejection fraction in a multicenter cohort."* The study represents a valuable contribution to digital health, demonstrating how domain-tuned LLMs can bridge diagnostic gaps between metabolic-associated fatty liver disease (MAFLD) and heart failure with preserved ejection fraction (HFpEF). The data are compelling, the writing is generally clear, and the clinical implications are significant.

Below are a set of **minor but necessary revisions** to ensure compliance with *PLOS Digital Health* standards and improve clarity and consistency. None of these affect the core analyses or conclusions.

1. Data Availability and Transparency (Policy Compliance)

To comply with the PLOS data policy, please make a small portion of the dataset *publicly available* in fully de-identified form, while keeping detailed patient-level data available upon request.

Required Actions:

- Deposit *fully de-identified, minimal underlying data* supporting all figures and tables (e.g., group-level summary statistics, confusion matrices, survival curve values) to a public repository such as OSF or Zenodo.
- Keep sensitive patient-level data under restricted access, available upon IRB-approved request.
- Harmonize the statements across the manuscript:
    - Replace conflicting versions on **page 5** and **page 20** with:

      "De-identified numerical data underlying the figures and tables are publicly available at [repository DOI link]. Access to full patient-level data requires approval from the Institutional Review Board of Peng Cheng Laboratory (contact: 15896850171@163.com)."

Suggested minimal uploads:

- table1_summary.csv – baseline variables (means, SDs, counts, percentages)
- table2_metrics.csv – model performance metrics and 2×2 confusion matrix
- figure2_survival.csv – all-cause and CV mortality (time, survival, 95% CI)
- figure3_rehospitalization.csv – rehospitalization rates and ORs
- prompt_and_threshold.txt – full standardized prompt and classification threshold
- readme_data_dictionary.txt – definition of each variable

These minimal datasets should fulfill reproducibility expectations without breaching privacy.

2. Terminology Clarifications

- **Abstract (p. 8–9):** Replace “while excluding imaging” with:

  “Raw imaging data were excluded; structured echocardiographic parameters and imaging report text were included.”

- **Methods (p. 15):** The subheading *“Prospective Validation”* could mislead readers. Revise to:

  “Blinded validation of prospectively sampled MedGuide-identified cases.”

3. Formatting and Style Adjustments

- Ensure all figures (especially Figures 2–3) are submitted as high-resolution, vector-based graphics (PDF/SVG). Maintain the number-at-risk table beneath survival curves.
- In Table 1, standardize units (“mL,” “mmHg,” “bpm”) and confirm uniform use of *P*-value symbols: † for P < 0.001, * for P < 0.05.
- Replace non-ASCII punctuation marks throughout (e.g., “，” → “,”; “：” → “:”).

4. Typos, Formatting, and Micro-Edits

Below are itemized corrections, grouped by section. These should be implemented directly to improve polish and readability.

Global Corrections

- Replace all instances of full-width punctuation (e.g., “，”, “：”) with standard punctuation.
- Normalize percentage formatting: remove spaces before the % sign (e.g., 17.6 % → 17.6%).
- Use consistent notation for model names: “ChatGPT-4,” “Llama 3.1 (405B),” “MedGuide-14B.”

Title Page & Affiliations (pp. 6–7)

- Combine redundant institutional names (e.g., “Cardiovascular Research Institute Maastricht” appears twice).
- Standardize capitalization: “Maastricht, The Netherlands” (not “MAASTRICHT, NETHERLANDS, KINGDOM OF THE”).
- "Henan Provincial People's Hospital 锛 Zhengzhou University People's

Hospital" "Henan Provincial People's Hospital, Zhengzhou University People's Hospital".

Abstract (pp. 8–9)

- "fine-tuned on largescale clinical encounters" → "fine-tuned on large-scale clinical encounters."
- Ensure consistent use of commas and spacing for percentages: 12.7%, 17.6%, 90.4%.
- Add article before model name: "The MedGuide-14B model substantially increased detection…"

Methods (pp. 10–14)

- "forementioned Ethics Committees" → "aforementioned Ethics Committees."
- "EHRs data" → "EHR data."
- Use consistent capitalization for variable names: "H2FPEF" and "HFA-PEFF."
- Clarify imputation: "Imputed missing laboratory and symptom data using median and mode imputation."
- Replace "chain-of-thought decomposition" with "chain-of-thought reasoning."
- Standardize LVEF expression: "LVEF ≥ 50%."

Results (pp. 15–16)

- "Prospective sample" section: change to "Blinded validation subset."
- Include n/N explicitly for clarity (optional): e.g., "55.6% (2,351/4,226)."
- Verify unit consistency (mm, mL, %).
- Replace "non-confirmed" with "nonconfirmed" for consistency.

Discussion (pp. 17–20)

- Correct "gap of HFpEF diagnosis" → "gap in HFpEF diagnosis."
- "24 011" → "24,011."
- "17.6 %" → "17.6%."
- "ChatGPT 4.0" → "ChatGPT-4."
- "and, and future work" → "and future work."
- Standardize punctuation around model comparisons: "Llama 3.1 (405B)" (no missing parentheses).
- When referencing prior studies, ensure uniform reference superscripts (e.g., ^{32} → [32]).

Data Availability & Funding (p. 20)

- Replace full-width colon: “Data Availability Statement：” → “Data Availability Statement:”
- Rephrase the paragraph to reflect revised policy-compliant language (see Section 1).

Figures and Tables (pp. 21–29)

- Remove embedded file names from figure captions (e.g., “Figure 1 .jpg”).
- Ensure color palettes are colorblind-friendly and line thickness consistent across panels.
- Figure 2: align legend order with curve order and keep number-at-risk visible.
- Figure 3: ensure axes are labeled “Rehospitalization Rate (%)” and “Odds Ratio.”

References (pp. 24–26)

- Standardize reference style: Author(s). Title. *Journal Name* Year; Volume(Issue): Page–Page.
- Ensure consistency in journal title capitalization (e.g., *Eur Heart J Digit Health* not *European Heart J Digit Health* if journal style requires abbreviation).
- Cross-check the 2016 ESC guideline reference (Ref 18) and ensure correct citation format per PLOS style.

5. Language and Readability

Overall language quality is high. Please consider the following optional refinements:

- Replace “the forementioned” → “the aforementioned.”
- Replace “LLMs bridge the gap of HFpEF diagnosis” → “LLMs can bridge the diagnostic gap in HFpEF.”
- Replace “non-confirmed” → “nonconfirmed” (consistency).
- Prefer concise, formal phrasing (e.g., “This highlights” rather than “This clearly highlights”).
