# Supplementary material for: Large language model detects previously undiagnosed heart failure with preserved ejection fraction in patients with metabolic-associated fatty liver disease: A multicenter cohort study
Source: PLOS Digit Health. 2026 Mar 31;5(3):e0001317. doi: 10.1371/journal.pdig.0001317 (PMC13037960; doi:10.1371/journal.pdig.0001317)
Supplement: Attachment — Submitted filename: 2.Response to Reviewers-2026.1.26.docx [file pdig.0001317.s007.docx]

**Response to the Reviewer and Reviewers**

**Manuscript ID: PDIG-D-25-00681**

We sincerely appreciate the Reviewer and the Reviewers for their careful evaluation and constructive comments on our manuscript. In response, we address each comment individually and provide detailed explanations to clarify the corresponding revisions. To facilitate the review process, each response is followed by a concise and explicit summary of the related modifications, with precise references to the relevant sections of the manuscript. All revisions have been incorporated into the revised version and are highlighted accordingly.

Given the length of this response, we provide a table of contents below to facilitate navigation.

**Table of Contents**

[Editor Comments	1](#_Toc220223531)

[Reviewer #1	9](#_Toc220223532)

[Reviewer #2	28](#_Toc220223533)

[Reviewer #3	33](#_Toc220223534)

# **Editor Comments**

**The study is innovative and aligns closely with the journal’s aims, but it requires enhanced methodological transparency, calibration analysis, data openness, and clearer articulation of clinical implications. The results are promising yet preliminary without external validation or model interpretability evaluation. Addressing these issues would elevate the paper to a publishable standard.**

**Response:** We sincerely thank the Editor for the insightful and constructive summary of the manuscript’s strengths and areas requiring further clarification. In response to these comments, we have undertaken a comprehensive revision focused on improving methodological transparency, calibration assessment, data openness, and the clarity of clinical interpretation.

We believe these revisions directly address the Editor’s concerns and substantially strengthen the methodological rigor, interpretability, and clinical relevance of the manuscript.

**Methodology**

**Strengths**

**• Well-defined inclusion/exclusion criteria based on ESC HFpEF diagnostic criteria.**

**• Appropriate selection of performance metrics (AUC, PPV, NPV, sensitivity, specificity).**

**• Clinical adjudication by cardiologists enhances reliability.**

**Response:** We thank the Editor for the positive assessment of the methodological strengths of our study. We appreciate the recognition of the well-defined inclusion and exclusion criteria based on ESC HFpEF diagnostic standards, the appropriate selection of clinically relevant performance metrics, and the use of cardiologist adjudication to enhance the reliability of outcome assessment. These elements were intentionally incorporated to ensure methodological rigor and clinical relevance.

**Concerns / Suggestions**

**• Clarify model architecture and training: Was MedGuide-14B trained exclusively on Chinese EHR data, and was it fine-tuned using domain-specific corpora (e.g., cardiology notes)?**

**Response:** Thank you for raising this important point. In the revised manuscript, we clarified that the primary contribution of this study is the evaluation of an existing domain-aligned large language model (MedGuide-14B) for HFpEF identification, rather than the development or full pretraining of a new model.

We therefore describe the model architecture and training background to support reproducibility and transparency of inference in this study. Specifically, MedGuide-14B was developed through prior domain adaptation using heterogeneous, de-identified clinical corpora spanning multiple medical specialties, and was applied to the evaluation cohorts in a frozen, inference-only setting.

Importantly, no additional training, fine-tuning, or parameter updating was performed on any data from the participating centers or evaluation cohorts, thereby avoiding information leakage. The model was applied in a strictly frozen, inference-only manner throughout all analyses.

To prevent overinterpretation, we clarify that the present study focuses on the clinical application of a domain-aligned large language model, rather than on benchmarking the effects of specific pretraining data sources (e.g., country- or specialty-specific corpora).

**Changes made in the manuscript:**

Methods-expanded the description of MedGuide-14B model background and inference-only usage, explicitly clarifying the scope of training information provided and stating that no fine-tuning or parameter updating was performed on evaluation cohorts (Page 9, Lines 196–218).

**• Describe training–validation–test data splits and strategies to prevent data leakage.**

**Response:** We agree and have clarified the dataset partitioning strategy and leakage-prevention measures. In the revised Methods, we explicitly describe how cohorts were separated for model evaluation, and we added safeguards to prevent overlap and information leakage across splits (e.g., patient-level separation, time window handling where applicable, and consistent application of a fixed threshold). We also stated that the model was invoked in an inference-only manner for the evaluation cohorts, without any parameter updates.

**Changes made in the manuscript:**

Methods-added/expanded details on study design, data sources, and evaluation setup including split strategy and leakage prevention (Page 7, Lines 149–155; Page 10, Lines 233–240; Page 9, Lines 196–218).

**• Provide calibration performance (e.g., Brier score, calibration plots) to complement discrimination metrics.**

**Response:** We appreciate this suggestion and have added calibration analyses accordingly, including the Brier score and decile-based calibration assessment.

**Changes made in the manuscript:**

Methods-added a new subsection describing calibration analysis (Brier score and calibration approach) (Page 11, Lines 258–263).

Results-added a dedicated paragraph reporting calibration performance (Page 14, Lines 344–348).

Data Availability Statement-Zenodo released calibration underlying data (DOI: <https://doi.org/10.5281/zenodo.17970844>). (Page 19, Lines 475–483)

**• Discuss model interpretability: How are feature attributions or token contributions verified against clinical reasoning?**

**Response:** Thank you for highlighting interpretability. We have expanded the Discussion to clarify how interpretability was addressed in this study and to delineate the scope of the analyses performed. Given that MedGuide-14B operates on mixed structured and free-text EHR inputs, interpretability was approached through performance evaluation and blinded clinical adjudication. We further discuss practical interpretability-oriented strategies, such as structured rationale templates, clinician review of model-generated explanations, and targeted perturbation analyses, as important directions for future work.

**Changes made in the manuscript:**

Discussion-expanded interpretability considerations and limitations (Page 16, Lines 398–403; Page 16, Lines 405–410; Page 16–17, Lines 414–421).

Limitations – Interpretability limitation(Page 18, Lines 460–468).

**• Expand methods for handling missing or inconsistent EHR data.**

**Response:** We agree and have expanded the Methods to describe preprocessing steps for missing or inconsistent EHR entries. In the revised manuscript, we clarify how structured variables were standardized, how missingness was handled at the variable level, and how inconsistent entries were addressed through preprocessing and rule-based normalization prior to model invocation. These additions improve methodological transparency and facilitate replication.

**Changes made in the manuscript:** Methods-expanded descriptions of data preprocessing and missing data handling (Page 9–10, Lines 220–231; Page 10, Lines 232–237).

**• Consider external validation or cross-site holdout testing to confirm generalizability.**

**Response:** We appreciate this important suggestion regarding generalizability. We agree that external validation or cross-site holdout testing is essential for establishing the robustness and clinical transferability of large language model–based approaches.

In the present study, our primary objective was to evaluate the feasibility and clinical relevance of applying a domain-tuned large language model to identify previously unrecognized HFpEF within real-world EHR data, rather than to provide definitive external validation. Although data were collected from multiple centers, differences in EHR structure, documentation practices, and data governance policies limited the feasibility of conducting a fully independent external validation or strict cross-site holdout analysis within the current retrospective framework.

To address this limitation transparently, we have explicitly acknowledged the absence of external validation in the Discussion section and emphasized that the findings should be considered exploratory. We further state that prospective validation and external testing across diverse healthcare systems and populations will be a critical next step before broader clinical deployment.

**Changes made in the manuscript:**

Discussion-expanded the limitations subsection to explicitly acknowledge the lack of external validation and to outline the need for future prospective and cross-site validation studies to confirm generalizability (Page 18, Lines 460–468).

**Data**

**The data-availability statement limits access to “reasonable request,” which is non-compliant with PLOS open-data policy. Derived or anonymized data, synthetic EHRs, and reproducible model code should be made publicly accessible to support transparency. I recommend providing:**

**• De-identified or synthetic EHR datasets sufficient for replication.**

**Response:** We thank the Editor for highlighting the importance of data transparency and compliance with the PLOS open-data policy. We have revised the Data Availability Statement accordingly and deposited a comprehensive set of fully de-identified, group-level numerical data in a public Zenodo repository.

The publicly available data support reproduction of all primary analyses, figures, and tables, including baseline characteristics, model performance metrics, blinded clinician validation results, survival curve values, rehospitalization outcomes, calibration analyses, and standardized inference prompts.

**Changes made in the manuscript:** Data Availability Statement-revised to remove the “reasonable request” restriction and to provide a permanent public repository link to de-identified numerical data supporting all figures and tables (Page 19, Lines 476–482). The Zenodo repository has been added with DOI: https://doi.org/10.5281/zenodo.17970844.

**• Model code, prompts, or weights on a public platform (e.g., GitHub, Zenodo).**

**Response:** We thank the Editor for this suggestion regarding reproducibility and transparency. We have made the core components required to reproduce the inference workflow publicly available, including standardized inference prompts, probability threshold definitions, and de-identified numerical data, all deposited in a public Zenodo repository.

The MedGuide-14B model is publicly accessible via an established open-source model hub (Hugging Face) and was applied using a fixed, inference-only configuration consistent with the specifications shared in this study.

**Changes made in the manuscript:** Data Availability Statement-expanded to explicitly reference the public availability of standardized inference prompts and threshold specifications in the Zenodo repository, and to clarify that MedGuide-14B model weights are publicly accessible via Hugging Face and were used in a frozen, inference-only manner (Page 19, Lines 476–483).

**• Example inference pipeline demonstrating reproducibility.**

**Response:** We appreciate this suggestion and agree that providing a clear inference pipeline is essential for reproducibility. In response, we have explicitly documented the end-to-end inference workflow used in this study and made the key components publicly available.

Specifically, the publicly deposited materials include (1) standardized inference prompts, (2) explicit probability threshold definitions for HFpEF classification, and (3) fully de-identified numerical outputs underlying all reported analyses. Together, these elements define a reproducible inference pipeline in which electronic health record data are structured, parsed, and analyzed by MedGuide-14B in a frozen, inference-only manner to generate HFpEF probability estimates and supporting evidence.

In addition, we describe the inference process step by step in the Methods section, including data preprocessing, model invocation, probability estimation, threshold-based classification, and downstream statistical analysis. This documentation enables other researchers to replicate the analytical workflow using the same model and prompts, without requiring access to restricted patient-level data.

**Changes made in the manuscript:**

Methods-expanded the descriptions of data processing, inference strategy, and probability threshold selection to explicitly delineate the end-to-end inference pipeline (Page 9, Lines 220–231; Page 10, Lines 233–237; Page 10, Lines 239–252).

Data Availability Statement-updated to reference the public Zenodo repository containing standardized inference prompts, threshold definitions, and example model outputs supporting reproducibility (Page 19, Lines 476–483).

# **Reviewer #1**

**Thank you for submitting this well-prepared and clinically relevant manuscript, “Large language model detects previously undiagnosed heart failure with preserved ejection fraction in a multicenter cohort.” The study represents a valuable contribution to the field of digital health, demonstrating how domain-tuned large language models can help bridge diagnostic gaps between metabolic-associated fatty liver disease (MAFLD) and heart failure with preserved ejection fraction (HFpEF). The data are compelling, the writing is generally clear, and the clinical implications are significant.**

**Below are a set of minor but necessary revisions to ensure compliance with PLOS Digital Health standards and improve clarity and consistency. None of these affect the core analyses or conclusions.**

**Response:** We sincerely thank Reviewer #1 for the positive evaluation of our work and for the detailed and constructive suggestions. We have carefully addressed all comments to improve clarity, transparency, and compliance with PLOS Digital Health standards, as detailed below.

**Comment 1: Data Availability and Transparency**

**To comply with the PLOS data policy, please make a small portion of the dataset publicly available in fully de-identified form, while keeping detailed patient-level data available upon request.**

**Required Actions:**

**• Deposit fully de-identified, minimal underlying data supporting all figures and tables (e.g., group-level summary statistics, confusion matrices, survival curve values) to a public repository such as OSF or Zenodo.**

**Response:** We thank the Reviewer for clearly outlining the requirements for compliance with the PLOS data policy. We have completed all requested steps and revised the manuscript accordingly.

A comprehensive set of fully de-identified, minimal underlying data supporting all figures and tables has been deposited in a public Zenodo repository. These materials include group-level baseline summaries, model performance metrics, blinded clinician validation results, survival curve values, rehospitalization outcomes, calibration data, and standardized inference prompts, enabling reproduction of all primary analyses and visualizations.

**Changes made in the manuscript:** Data Availability Statement-revised to explicitly reference the public Zenodo repository containing fully de-identified, minimal datasets supporting all figures and tables, and to clarify the conditions under which access to patient-level data may be requested (Page 19, Lines 476–483). The permanent repository link and DOI have been added: https://doi.org/10.5281/zenodo.17970844.

**• Keep sensitive patient-level data under restricted access, available upon IRB-approved request.**

**Response:** We agree with this requirement and have implemented it accordingly. Fully de-identified, minimal underlying numerical data supporting all reported analyses and figures have been made publicly available via a Zenodo repository.

Details regarding access to individual-level electronic health record data are described in the revised Data Availability Statement.

**Changes made in the manuscript:** The Data Availability Statement was revised to reflect public availability of de-identified numerical data via Zenodo (Page 19, Lines 476–483).

**• Harmonize the statements across the manuscript:**

**o Replace conflicting versions on page 5 and page 20 with:**

**“De-identified numerical data underlying the figures and tables are publicly available at [repository DOI link]. Access to full patient-level data requires approval from the Institutional Review Board of Peng Cheng Laboratory (contact:** [**15896850171@163.com)**](mailto:15896850171@163.com))**.”**

**Response:** We thank the Reviewer for identifying this inconsistency. We have carefully harmonized all data availability statements across the manuscript and replaced the conflicting versions with the exact wording recommended by the Reviewer.

The revised statement now appears consistently wherever data availability is described, ensuring clarity and alignment with PLOS Digital Health data policy requirements.

**Changes made in the manuscript:** Data Availability Statement-standardized and unified across the manuscript by replacing all conflicting versions with the following text: “De-identified numerical data underlying the figures and tables are publicly available at https://doi.org/10.5281/zenodo.17970844. Access to full patient-level data requires approval from the Institutional Review Board of Peng Cheng Laboratory (contact: [15896850171@163.com)](mailto:15896850171@163.com)).” (Page 19, Lines 476–483).

**Suggested minimal uploads:**

**• table1_summary.csv – baseline variables (means, SDs, counts, percentages)**

**• table2_metrics.csv – model performance metrics and 2×2 confusion matrix**

**• figure2_survival.csv – all-cause and cardiovascular mortality (time, survival, 95% CI)**

**• figure3_rehospitalization.csv – rehospitalization rates and odds ratios**

**• prompt_and_threshold.txt – full standardized prompt and classification threshold**

**• readme_data_dictionary.txt – definition of each variable**

**These minimal datasets should fulfill reproducibility expectations without breaching patient privacy.**

**Response**: We thank the Reviewer for providing this clear and practical guidance. We confirm that all suggested minimal datasets have been prepared and deposited in a public Zenodo repository, fully satisfying reproducibility requirements while preserving patient privacy.

Specifically, the Zenodo deposit includes: (1) group-level baseline summary statistics (table1_summary_completed.csv); (2) model performance metrics and confusion matrix–derived measures (table2_metrics_completed.csv); (3) Kaplan–Meier survival curve values for all-cause and cardiovascular mortality (figure2_survival.csv); (4) rehospitalization outcomes with unadjusted relative risks and odds ratios (figure3_rehospitalization_inferred.csv); (5) the full standardized inference prompt and HFpEF classification threshold (prompt_and_threshold.txt); and (6) a detailed data dictionary describing all variables and file contents (readme.txt).

All files are fully de-identified and contain no patient-level identifiers. Together, these datasets enable independent reproduction of all figures, tables, and primary analyses reported in the manuscript without breaching ethical or privacy constraints.

**Changes made in the manuscript**: Data Availability Statement-updated to explicitly reference the publicly deposited minimal datasets supporting reproducibility, with a permanent Zenodo link and DOI (https://doi.org/10.5281/zenodo.17970844

) (Page 19, Lines 476–483).

**2. Terminology Clarifications**

**• Abstract (p. 8–9): Replace “while excluding imaging” with: “Raw imaging data were excluded; structured echocardiographic parameters and imaging report text were included.”**

**Response**: We thank the Reviewer for this helpful clarification. To avoid ambiguity regarding the use of imaging-related information, we have revised the wording in the Abstract exactly as suggested.

The updated text now clearly distinguishes between the exclusion of raw imaging data and the inclusion of structured echocardiographic parameters and imaging report text, ensuring accurate representation of the data sources used in this study.

**Changes made in the manuscript**:

Abstract-replaced the phrase “while excluding imaging” with “Raw imaging data were excluded; structured echocardiographic parameters and imaging report text were included” to clarify data usage (Page 3, Lines 58–67).

**• Methods (p. 15): The subheading “Prospective Validation” could mislead readers. Revise to: “Blinded validation of prospectively sampled MedGuide-identified cases.”**

**Response:** We thank the Reviewer for pointing out this potentially misleading wording. To ensure clarity and to accurately reflect the study design, we have revised the subheading exactly as suggested.

The updated subheading now clearly indicates that the validation was blinded and conducted on prospectively sampled MedGuide-identified cases, rather than representing a fully prospective study.

**Changes made in the manuscript:** Methods-revised the subheading “Prospective Validation” to “Blinded validation of prospectively sampled MedGuide-identified cases” to avoid misinterpretation of the study design (Page 13, Lines 328–329).

**3. Formatting and Style Adjustments**

**• Ensure all figures (especially Figures 2–3) are submitted as high-resolution, vector-based graphics (PDF/SVG). Maintain the number-at-risk table beneath survival curves.**

**Response:** We thank the Reviewer for this guidance regarding figure quality and presentation standards. We confirm that all figures, including Figures 2 and 3, have been regenerated and submitted as high-resolution, vector-based graphics in PDF format to ensure optimal clarity and scalability for publication.

In addition, the number-at-risk tables beneath the Kaplan–Meier survival curves have been retained as requested, preserving the full interpretability of the time-to-event analyses.

**Changes made in the manuscript:** Figures 2 and 3-updated and resubmitted as high-resolution, vector-based PDF files, with number-at-risk tables maintained beneath the survival curves, in accordance with PLOS Digital Health figure requirements.

**• In Table 1, standardize units (“mL,” “mmHg,” “bpm”) and confirm uniform use of P-value symbols: † for P < 0.001, * for P < 0.05.**

**Response:** We thank the Reviewer for this careful review of table formatting and statistical notation. We have standardized all measurement units in Table 1, including the consistent use of “mL,” “mmHg,” and “bpm,” and have carefully reviewed the table to ensure uniform and correct application of P-value symbols.

Specifically, the dagger symbol (†) is now used consistently to denote P < 0.001, and the asterisk (*) is used to denote P < 0.05 throughout Table 1.

**Changes made in the manuscript:** Table 1-units standardized across all variables and P-value symbols harmonized, with † indicating P < 0.001 and * indicating P < 0.05, as specified by the Reviewer.

**• Replace non-ASCII punctuation marks throughout (e.g., “，” → “,”; “：” → “:”).**

**Response:** We thank the Reviewer for this formatting reminder. We have carefully reviewed the entire manuscript and replaced all non-ASCII punctuation marks with standard ASCII equivalents, including commas, colons, and other punctuation, to ensure consistency and compatibility with journal production requirements.

**Changes made in the manuscript:** Manuscript-wide-replaced all non-ASCII punctuation marks with standard ASCII punctuation throughout the text, tables, and figure captions.

**4. Typos, Formatting, and Micro-Edits**

**Below are itemized corrections, grouped by section. These should be implemented directly to improve polish and readability. Global Corrections**

**• Replace all instances of full-width punctuation (e.g., “，”, “：”) with standard punctuation.**

**Response:** We thank the Reviewer for this detailed formatting guidance. As requested, we have implemented this correction globally across the manuscript. All instances of full-width punctuation have been systematically replaced with standard ASCII punctuation to improve consistency, readability, and compatibility with journal production standards.

**Changes made in the manuscript:** Manuscript-wide-replaced all full-width punctuation marks with standard punctuation throughout the text, tables, figure captions, and supplementary materials.

**• Normalize percentage formatting: remove spaces before the % sign (e.g., 17.6 % → 17.6%).**

**Response:** We thank the Reviewer for this formatting suggestion. We have reviewed the entire manuscript and normalized percentage formatting throughout by removing spaces before the percent sign, ensuring consistent presentation in accordance with journal style guidelines.

**Changes made in the manuscript:** Manuscript-wide-normalized percentage formatting by removing spaces before the % sign throughout the text, tables, figure captions, and supplementary materials.

**• Use consistent notation for model names: “ChatGPT-4,” “Llama 3.1 (405B),” “MedGuide-14B.”**

**Response:** We thank the Reviewer for this helpful suggestion. We have carefully reviewed the manuscript and standardized the notation of all model names to ensure consistency and clarity. Specifically, the model names “ChatGPT-4,” “Llama 3.1 (405B),” and “MedGuide-14B” are now used uniformly throughout the text.

**Changes made in the manuscript:** Manuscript-wide-standardized the notation of model names, ensuring consistent use of “ChatGPT-4,” “Llama 3.1 (405B),” and “MedGuide-14B” across the text, tables, figure captions, and supplementary materials.

**Title Page & Affiliations (pp. 6–7)**

**• Combine redundant institutional names (e.g., “Cardiovascular Research Institute Maastricht” appears twice).**

**Response:** We thank the Reviewer for identifying this redundancy. We have carefully reviewed the Title Page and affiliation list and combined duplicate institutional names to eliminate repetition, while preserving accurate author–institution associations.

No institutional affiliations were removed or altered in substance; the revisions were made solely to improve clarity, conciseness, and consistency in presentation.

**Changes made in the manuscript:** Title Page & Affiliations-merged redundant institutional names and removed duplicated entries to ensure a concise and consistent affiliation list (Page 1–2, Lines 10–35).

**• Standardize capitalization: “Maastricht, The Netherlands” (not “MAASTRICHT, NETHERLANDS, KINGDOM OF THE”).**

**Response:** We thank the Reviewer for this formatting correction. We have standardized capitalization for all geographic names and institutional locations throughout the manuscript, including the use of “Maastricht, The Netherlands,” to ensure consistency and compliance with journal style guidelines.

**Changes made in the manuscript:** Title Page & Affiliations-standardized capitalization and formatting of geographic names and locations, including revision to “Maastricht, The Netherlands” (Page 1–2, Lines 10–35).

**• "Henan Provincial People's Hospital & Zhengzhou University People's Hospital" "Henan Provincial People's Hospital, Zhengzhou University People's Hospital".**

**Response:** Thank you for the formatting suggestion. The institutional name has been revised accordingly.

**Changes made in the manuscript:** Title Page & Affiliations-revised the institutional name to “Henan Provincial People's Hospital, Zhengzhou University People's Hospital” by replacing the ampersand with a comma (Page 1–2, Lines 10–35).

**Abstract (pp. 8–9)**

**• “fine-tuned on largescale clinical encounters” → “fine-tuned on large-scale clinical encounters.”**

**Response:** We thank the Reviewer for noting this typographical issue. We have corrected the wording as suggested to ensure proper spelling and consistency with standard scientific usage.

**Changes made in the manuscript:** Abstract-corrected “largescale” to “large-scale” in the description of model training (Page 3, Lines 58).

**• Ensure consistent use of commas and spacing for percentages: 12.7%, 17.6%, 90.4%.**

**Response:** We thank the Reviewer for this formatting clarification. We have reviewed the manuscript and ensured consistent formatting of percentages throughout, including the correct use of commas and the removal of spaces before the percent sign (e.g., 12.7%, 17.6%, 90.4%).

**Changes made in the manuscript:** Manuscript-wide-standardized percentage formatting to ensure consistent use of commas and spacing for all percentages across the text, tables, figure captions, and supplementary materials.

**• Add article before model name: “The MedGuide-14B model substantially increased detection…”**

**Response:** We thank the Reviewer for this language refinement. We have revised the sentence as suggested by adding the definite article before the model name to ensure grammatical correctness and improve readability.

**Changes made in the manuscript:** Manuscript-wide-added the definite article “the” before the model name “MedGuide-14B” where appropriate to ensure consistent and grammatically correct usage.

**Methods (pp. 10–14)**

**• “forementioned Ethics Committees” → “aforementioned Ethics Committees.” • “EHRs data” → “EHR data.”**

**Response:** We thank the Reviewer for identifying these language issues. We have revised the wording as suggested to ensure grammatical correctness and consistency with standard scientific usage.

**Changes made in the manuscript:**

Methods-replaced “forementioned Ethics Committees” with “aforementioned Ethics Committees” and corrected “EHRs data” to “EHR data” throughout the Methods section (Page 8, Lines 166–167; Page 9, Lines 220).

**• Use consistent capitalization for variable names: “H2FPEF” and “HFA-PEFF.”**

**Response:** We thank the Reviewer for this clarification. We have reviewed the manuscript and standardized the capitalization of diagnostic score names to ensure consistent use of “H2FPEF” and “HFA-PEFF” throughout the text.

**Changes made in the manuscript:** Manuscript-wide-standardized the capitalization of the diagnostic score names “H2FPEF” and “HFA-PEFF” across the text, tables, figure captions, and supplementary materials.

**• Clarify imputation: “Imputed missing laboratory and symptom data using median and mode imputation.”**

**Response:** We thank the Reviewer for this request for clarification. We have revised the Methods section to explicitly state the imputation strategy used for missing data. As clarified, missing laboratory variables were imputed using the median, and missing categorical or symptom variables were imputed using the mode.

This revision improves methodological transparency without altering the analytical approach or results.

**Changes made in the manuscript:** Methods-clarified the handling of missing data by explicitly stating that missing laboratory values were imputed using median imputation and missing symptom or categorical variables using mode imputation (Page 10, Lines 233–237).

**• Replace “chain-of-thought decomposition” with “chain-of-thought reasoning.”**

**Response:** We thank the Reviewer for this terminology clarification. We have revised the wording as suggested by replacing “chain-of-thought decomposition” with “chain-of-thought reasoning” to ensure accurate and standard usage of terminology.

**Changes made in the manuscript:** Methods-replaced “chain-of-thought decomposition” with “chain-of-thought reasoning” to standardize terminology and improve clarity (Page 9, Lines 197–213).

**• Standardize LVEF expression: “LVEF ≥ 50%.”**

**Response:** We thank the Reviewer for this clarification. We have reviewed the manuscript and standardized the expression of left ventricular ejection fraction to “LVEF ≥ 50%” throughout, ensuring consistent and clear presentation.

**Changes made in the manuscript:** Manuscript-wide-standardized the expression of left ventricular ejection fraction as “LVEF ≥ 50%” across the text, tables, figure captions, and supplementary materials.

**Results (pp. 15–16)**

• **“Prospective sample” section: change to “Blinded validation subset.”**

**Response:** Thank you for this helpful suggestion. To avoid potential misunderstanding regarding study design, we have revised the terminology in the Results section to reflect the actual evaluation setting. The analyzed cohort represents a blinded validation subset of MedGuide-identified cases rather than a prospective sample.

**Changes made in the manuscript:** Results section-revised the subsection title and corresponding text from “Prospective sample” to “Blinded validation subset” to accurately describe the study design.( Page 13, Lines 323–329)

**• Include n/N explicitly for clarity (optional): e.g., “55.6% (2,351/4,226).”**

**Response:** Thank you for this suggestion. To improve clarity and interpretability, we have added explicit numerator/denominator information (n/N) alongside percentage values for key outcome measures, including rehospitalization and mortality outcomes, in the Results section.

**Changes made in the manuscript:** Results section-added explicit n/N values alongside percentages for rehospitalization and mortality outcomes enhance transparency and readability.( Page 12, Lines 298–310)

**• Verify unit consistency (mm, mL, %).**

**Response:** Thank you for pointing this out. We carefully reviewed all tables and figures to ensure consistency and correctness of measurement units throughout the manuscript. Units are now uniformly reported using standard SI conventions, including millimeters (mm) for linear dimensions, milliliters (mL) for volumes, and percentages (%) for proportions.

**Changes made in the manuscript:** Table 1 and associated text-verified and standardized all units (mm, mL, %, mmHg, bpm) to ensure consistent formatting across variables and sections.( Page 23–24, Lines 612–620)

**• Replace “non-confirmed” with “nonconfirmed” for consistency.**

**Response:** Thank you for noting this stylistic inconsistency. We have standardized the terminology throughout the manuscript by replacing “non-confirmed” with “nonconfirmed” to ensure consistent spelling and formatting.

**Changes made in the manuscript:** Table 3 and corresponding text in the Results section-all instances of “non-confirmed” have been revised to “nonconfirmed.” (Page 25, Lines 624)

**Discussion (pp. 17–20)**

**• Correct “gap of HFpEF diagnosis” → “gap in HFpEF diagnosis.”**

**• “24 011” → “24,011.”**

**• “17.6 %” → “17.6%.”**

**• “ChatGPT 4.0” → “ChatGPT-4.”**

**• “and, and future work” → “and future work.”**

**Response:** Thank you for identifying these typographical, formatting, and terminology inconsistencies. We have carefully revised the Discussion section to correct grammatical usage, standardize numerical and percentage formatting, ensure consistent model naming, and remove redundant wording. These edits were implemented to improve clarity, readability, and adherence to journal style guidelines, without altering the scientific content or interpretation.

**Changes made in the manuscript:** Discussion section:

Revised “gap of HFpEF diagnosis” to “gap in HFpEF diagnosis.”

Standardized numeric formatting from “24 011” to “24,011.”

Removed spacing in percentage notation (“17.6 %” → “17.6%”).

Standardized model naming to “ChatGPT-4.”

Corrected duplicated conjunction (“and, and future work” → “and future work”).( Page 14–16)

**• Standardize punctuation around model comparisons: “Llama 3.1 (405B)” (no missing parentheses).**

**Response:** Thank you for pointing out this punctuation inconsistency. We have standardized the formatting of model names in all model comparison statements to ensure correct use of parentheses and uniform presentation across the manuscript. This revision improves readability and prevents potential ambiguity when referring to specific model variants.

**Changes made in the manuscript:** Throughout the manuscript (Abstract, Introduction, Discussion, and Supplementary sections where applicable):

Corrected and standardized model name formatting to “Llama 3.1 (405B)” with complete parentheses and consistent typography.

**• When referencing prior studies, ensure uniform reference superscripts (e.g., ³² → [32]).**

**Response:** Thank you for highlighting this formatting issue. We have standardized all in-text citations throughout the manuscript by replacing superscript-style references with bracketed numerical citations (e.g., [32]) to ensure consistency with the journal’s reference formatting requirements.

**Changes made in the manuscript:** Throughout the manuscript (Abstract, Introduction, Methods, Results, Discussion, and Supplementary sections):

All superscript citation formats (e.g., ³²) have been revised to bracketed numerical references (e.g., [32]).

**Data Availability & Funding (p. 20)**

**• Replace full-width colon: “Data Availability Statement：” → “Data Availability Statement:”**

**• Rephrase the paragraph to reflect revised policy-compliant language (see Section 1).**

**Response:** Thank you for this clarification. We have corrected the punctuation by replacing the full-width colon with a standard ASCII colon and have revised the entire Data Availability Statement to fully comply with PLOS Digital Health open-data policies. The updated statement now clearly distinguishes between publicly available de-identified numerical data and restricted patient-level data, providing an explicit repository link and DOI for the former while outlining the approval process required to access the latter.

**Changes made in the manuscript:** Data Availability Statement:

Replaced the full-width colon with a standard colon (“Data Availability Statement:”).

Rewrote the paragraph to state that de-identified numerical data underlying all figures and tables are publicly available via Zenodo (DOI: https://doi.org/10.5281/zenodo.17970844).

Clarified that access to full patient-level EHR data requires Institutional Review Board approval from Peng Cheng Laboratory, with contact information provided. ( Page 19, Lines 447–483)

**Figures and Tables (pp. 21–29)**

**• Remove embedded file names from figure captions (e.g., “Figure 1 .jpg”).**

**Response:** Thank you for pointing out this formatting issue. We have removed all embedded file names and file extensions (e.g., “.jpg”, “.png”) from the figure captions to ensure that captions are clean, publication-ready, and focused solely on describing the figure content, in accordance with PLOS Digital Health formatting standards.

**Changes made in the manuscript:** Figures 1–3 captions-deleted all embedded file names and file extensions (e.g., “Figure 1.jpg”, “Figure 2.png”) and retained only the figure number and descriptive caption text (Page 29).

**• Ensure color palettes are colorblind-friendly and line thickness consistent across panels.**

**• Figure 2: align legend order with curve order and keep number-at-risk visible.**

**• Figure 3: ensure axes are labeled “Rehospitalization Rate (%)” and “Odds Ratio.”**

**Response:** Thank you for these detailed and helpful suggestions regarding figure presentation. We have carefully revised Figures 2 and 3 to improve clarity, accessibility, and consistency. Specifically, we adopted colorblind-friendly color palettes and standardized line thickness and visual styling across all panels. In Figure 2, the legend order was realigned to match the order of the Kaplan–Meier curves, and the number-at-risk table beneath the survival curves was retained and clearly formatted. In Figure 3, axis labels were revised to explicitly indicate “Rehospitalization Rate (%)” and “Odds Ratio,” ensuring accurate interpretation of the displayed risk estimates. These revisions enhance readability and comply with PLOS Digital Health visualization guidelines.

**Changes made in the manuscript:** Figures 2 and 3-updated color schemes to colorblind-friendly palettes and standardized line thickness across panels; aligned legend order with curve order and preserved the number-at-risk table in Figure 2; and revised axis labels to “Rehospitalization Rate (%)” and “Odds Ratio” in Figure 3 (Page 29).

**References (pp. 24–26)**

**• Standardize reference style: Author(s). Title. Journal Name Year; Volume(Issue): Page–Page.**

**• Ensure consistency in journal title capitalization (e.g., Eur Heart J Digit Health not European Heart J Digit Health if journal style requires abbreviation).**

**• Cross-check the 2016 ESC guideline reference (Ref 18) and ensure correct citation format per PLOS style.**

**Response:** Thank you for highlighting these important formatting issues. We have carefully reviewed and revised all references to ensure full compliance with PLOS Digital Health citation style. Specifically, all references have been standardized to the format “Author(s). Title. Journal Name Year; Volume(Issue): Page–Page.” Journal titles have been harmonized using the appropriate standardized abbreviations and capitalization throughout the reference list. In addition, the 2016 ESC guideline (Reference 18) has been cross-checked against the original publication and reformatted to ensure accuracy and consistency with PLOS requirements.

**Changes made in the manuscript:** References-standardized all entries to the required PLOS citation format, harmonized journal title abbreviations and capitalization, and corrected and reformatted Reference 18 (2016 ESC Guidelines) to ensure accuracy and compliance with journal style (Page 20–23).

**5. Language and Readability**

**Overall language quality is high. Please consider the following optional refinements:**

**• Replace “the forementioned” → “the aforementioned.”**

**• Replace “LLMs bridge the gap of HFpEF diagnosis” → “LLMs can bridge the diagnostic gap in HFpEF.”**

**• Replace “non-confirmed” → “nonconfirmed” (consistency).**

**• Prefer concise, formal phrasing (e.g., “This highlights” rather than “This clearly highlights”).**

**Response:** Thank you for these helpful suggestions regarding language refinement and stylistic consistency. We have carefully revised the manuscript to address all listed points, improving clarity, conciseness, and formal academic tone throughout. Specific wording has been corrected for accuracy (“aforementioned”), terminology has been standardized (“nonconfirmed”), and several sentences have been streamlined to remove unnecessary intensifiers and enhance readability. These revisions were applied consistently across the manuscript.

**Changes made in the manuscript:** Methods & Ethical considerations-corrected “the forementioned” to “the aforementioned” (Page 7,lines 162-167).

Discussion-revised phrasing to “LLMs can bridge the diagnostic gap in HFpEF” (Page 15–17).

Results & Tables-standardized terminology from “non-confirmed” to “nonconfirmed” (Page 24).

Manuscript-wide-simplified phrasing to improve clarity and formal tone (e.g., “This highlights” instead of “This clearly highlights”).

# **Reviewer #2**

**Reviewer #2: This is an interesting study to evaluate the large language model MedGuide-14B for HFpEF detection. There are several suggestions to improve the manuscript:**

**Response:** We sincerely thank Reviewer #2 for the positive assessment of our study and for the constructive and detailed suggestions provided. In response to these comments, we have carefully revised the manuscript to improve clarity, methodological transparency, and presentation quality. Specifically, we revised the title to explicitly indicate the MAFLD study population, restructured the Methods section by adding a dedicated Study Design subsection, expanded the description of MedGuide-14B development and training background, clarified the rationale for probability threshold selection, detailed follow-up procedures, improved figure resolution and layout, and refined the presentation of tables in accordance with the reviewer’s recommendations. All suggested revisions have been fully addressed and incorporated into the revised manuscript.

**1.It cannot be seen from the title that the patient has MAFLD. The title needs to be revised.**

**Response:** We thank the reviewer for this important suggestion. We have revised the title to explicitly indicate that the study population consists of patients with metabolic-associated fatty liver disease (MAFLD), thereby improving clarity and ensuring that the study context is immediately apparent to readers.

**Changes made in the manuscript:** Title-revised to explicitly include “patients with metabolic-associated fatty liver disease (MAFLD)” (Page 1).

**2.The first paragraph of "Ethical considerations" is not about ethical issues; it is about study design or data collection. I suggest to add the “study design” section.**

**Response:** We thank the reviewer for this insightful comment. In response, we have restructured the Methods section to improve clarity and logical organization. Specifically, we added a dedicated Study design and data sources subsection to clearly describe the study design and data collection process. Correspondingly, the Ethical considerations section has been revised to focus exclusively on ethical approval, informed consent waiver, and compliance with the Declaration of Helsinki.

**Changes made in the manuscript:** Methods-added a new subsection titled “Study design and data sources” describing the retrospective multicenter cohort design and data extraction procedures (Page 7, Lines 149–155).

Ethical considerations-revised to remove study design–related content and to focus solely on ethical approval and consent waiver (Page 8, Lines 162–167).

**3.In the “MedGuide-14B development and evaluation” section, it is better to report the amount of training data used.**

**Response:** We thank the reviewer for this helpful suggestion. In the revised manuscript, we have expanded the description of MedGuide-14B development to explicitly report the scale of the training data used during domain alignment and fine-tuning. Specifically, we now state that MedGuide-14B was fine-tuned on more than 260,000 de-identified real-world clinical encounters spanning multiple medical specialties. This clarification improves transparency regarding the model’s training background while maintaining the focus of the study on clinical evaluation rather than model development.

**Changes made in the manuscript:** Methods-MedGuide-14B development and evaluation-added explicit reporting of the training data scale (“more than 260,000 de-identified real-world clinical encounters”) used for domain alignment and fine-tuning (Page 9, Lines 197–218).

**4.Why was the threshold of 0.7 selected to classify patients as HFpEF-positive? What is the basis for choosing to use 0.7?**

**Response:** We thank the reviewer for this important methodological question. In the revised manuscript, we have clarified that the probability threshold of 0.70 was selected based on performance optimization in a held-out validation dataset. Specifically, this threshold was chosen to balance sensitivity and specificity in alignment with the European Society of Cardiology (ESC) emphasis on sensitivity for HFpEF screening, where minimizing missed cases is clinically prioritized. At this threshold, MedGuide-14B achieved high sensitivity while maintaining acceptable specificity, supporting its suitability for identifying patients who warrant further cardiology evaluation rather than making definitive diagnoses.

**Changes made in the manuscript:** Methods-Patient classification and threshold selection-added a clear explanation of the rationale for selecting the 0.70 probability threshold, including validation-based optimization and alignment with ESC screening priorities (Page 10, Lines 239–245).

**5.Since this is a cohort study, the method needs to clearly specify how the follow-up is conducted, how often it is carried out, and what contents are being followed. It should not be simply written in a brief manner “Data were obtained via telephone follow-ups with patients or families and extracted from the electronic health record system (PIMS)”.**

**Response:** We thank the reviewer for this important comment. In response, we have substantially expanded the description of follow-up procedures to improve methodological clarity and transparency. The revised manuscript now explicitly specifies the follow-up modality (telephone interviews and EHR review), follow-up frequency, duration, and the clinical outcomes assessed. Follow-up was conducted at regular intervals through structured telephone contacts with patients or family members, complemented by systematic extraction of hospitalization and mortality data from the hospital electronic health record system (PIMS). The primary outcomes assessed during follow-up included rehospitalization events and all-cause mortality, with cardiovascular mortality adjudicated based on EHR documentation where available.

**Changes made in the manuscript:** Methods-Outcome data / Follow-up procedures-expanded the description of follow-up methods to specify follow-up modality, frequency, duration, and outcome contents, replacing the previous brief statement (Page 11, Lines 271–274).

**6.All figures need to be made clearer.**

**Response:** We thank the reviewer for this comment. In response, all figures have been carefully revised to improve clarity, readability, and consistency. Specifically, we enhanced figure resolution, optimized layouts, standardized fonts and line thickness, clarified axis labels and legends, and ensured colorblind-friendly color schemes. Number-at-risk tables were retained beneath survival curves where applicable. These revisions were made to facilitate accurate interpretation of the results and to align with PLOS Digital Health figure presentation standards.

**Changes made in the manuscript:** Figures 1–3-improved resolution and layout for all figures; standardized font size, line thickness, and color schemes across panels; clarified axis labels, legends, and annotations; and retained number-at-risk tables beneath Kaplan–Meier curves.

**7.Table 1: Did the Chi-square test consider the issue of multiple comparisons and perform Bonferroni adjustmen?**

**Response:** We thank the reviewer for raising this important statistical consideration. Table 1 is intended to provide a descriptive overview of baseline characteristics across study groups rather than to support confirmatory hypothesis testing. Therefore, no formal adjustment for multiple comparisons (e.g., Bonferroni correction) was applied. This approach is consistent with common practice in observational cohort studies, where baseline comparisons are presented descriptively to characterize the population. To improve transparency and avoid potential misinterpretation, we have explicitly clarified this rationale in the table footnote.

**Changes made in the manuscript:** Table 1 footnote-added a statement clarifying that baseline comparisons are descriptive in nature and that no formal adjustment for multiple comparisons was performed (Page 25).

**8.It is not recommended to divide Table 2 into (a) and (b) tables; instead, it is suggested to present it as two separate tables.**

**Response:** We thank the reviewer for this constructive suggestion. In response, we have revised the presentation of Table 2 by separating it into two independent tables to improve clarity and readability. One table now reports the diagnostic performance of MedGuide-14B for clinically diagnosed HFpEF, while the other presents the blinded validation results for MedGuide-identified HFpEF cases. This restructuring allows each table to convey a single, clearly defined message and aligns with standard table presentation practices.

**Changes made in the manuscript:** Tables-former Table 2(a) and Table 2(b) have been separated into Table 2 (Diagnostic performance of MedGuide-14B for clinically diagnosed HFpEF) and Table 3 (Blinded validation results for MedGuide-identified HFpEF cases). Table numbering, titles, and in-text citations have been updated accordingly (Page 26).

# **Reviewer #3**

**Reviewer #3: This paper investigates the application of MedGuide-14B, a domain-specific large language model, for the detection of heart failure with preserved ejection fraction (HFpEF) from electronic health records (EHRs) among patients with metabolic-associated fatty liver disease (MAFLD). MedGuide-14B is a purpose-built, open-source LLM, fine-tuned (with RLHF) on genuine multi-specialty medical dialogues and EHRs, not simply off-the-shelf.**

**Response:** We sincerely thank Reviewer #3 for the thoughtful summary of our work and for recognizing the domain-specific nature and clinical alignment of MedGuide-14B. In response to the reviewer’s comments, we have further clarified the primary contribution of this study-namely, the clinical application and evaluation of an existing domain-tuned large language model for HFpEF detection in a high-risk MAFLD population-rather than the development of a new foundation model. We have expanded the Methods section to provide clearer details regarding model training background, data preprocessing, prompt design, and inference setup, and enhanced the Discussion to address interpretability considerations, clinical relevance, and study limitations. In addition, we have improved reproducibility by making de-identified numerical data and standardized inference prompts publicly available and by providing clearer descriptions of how the model was invoked in this study.

**Changes made in the manuscript:**

Methods-MedGuide-14B development and evaluation-expanded description of model training background, fine-tuning strategy, data preprocessing, prompt design, and inference procedure (Page 9,lines 197-218).

Methods -clarified validation strategy and presentation of model outputs (Page11,lines254-257).

Discussion-expanded discussion on clinical interpretability, applicability, and limitations, including the scope of model generalizability (Page 15,lines363-383).

Data Availability Statement-added a public Zenodo repository link providing de-identified numerical data and standardized inference prompts to support reproducibility (Page 19,lines 476-477).

**I believe that the paper needs to further improve the following content to enhance its scientific influence:**

**1. More clearly indicate whether training and open-sourcing MedGuide-14B is a contribution of this paper, or whether applying MedGuide-14 to the detection of HFpEF is a contribution.**

**Response:** We thank the reviewer for this important clarification request. In the revised manuscript, we now explicitly state that the primary contribution of this study lies in the clinical application and evaluation of an existing, domain-tuned large language model (MedGuide-14B) for HFpEF detection in a high-risk MAFLD population, rather than in the training or open-sourcing of the model itself.

MedGuide-14B was developed and released prior to this study, and its architecture, training framework, and open-source availability are described only to the extent necessary to ensure transparency and reproducibility of the inference procedures used herein. We have revised the Abstract, Introduction, and Methods to clearly position this work as a clinical validation and real-world application study, focusing on disease underdiagnosis, prognostic relevance, and workflow integration, rather than as a model development paper.

**Changes made in the manuscript:**

Abstract: Clarified the study focus on the clinical application of MedGuide-14B for HFpEF detection (Page 3,lines 53-56).

Introduction: Framed the contribution as a clinical application and evaluation of a domain-tuned LLM in a MAFLD cohort (Page 6,lines131-139).

Methods: Clarified the background of MedGuide-14B development and open-source availability (Page 9,lines197-218).

**2. If considering the training and open-sourcing of MedGuide-14B as the main contribution, please supplement more detailed training details.The description of MedGuide-14B remains quite high-level. Please provide clearer details on fine-tuning, data preprocessing (for both structured variables and free text), prompt design, and inference setup.**

**Response:** We thank the Reviewer for this detailed and constructive request. To improve transparency and reproducibility of the clinical application reported in this study, we have expanded the methodological description of MedGuide-14B to an application-focused level.

Specifically, we added details on the fine-tuning strategy (supervised fine-tuning followed by reinforcement learning with human feedback), preprocessing of structured EHR variables and free-text clinical notes, standardized prompt design for HFpEF identification, and the inference setup used during evaluation.

**Changes made in the manuscript:**

Methods-MedGuide-14B development and evaluation-added details on supervised fine-tuning and RLHF-based medical alignment; expanded descriptions of preprocessing for structured EHR variables and free-text clinical notes; explicitly described the standardized prompt used for HFpEF screening; and clarified the inference setup, including frozen-model, inference-only usage and probability score generation (Page 9,lines197-218).

**3. I would encourage the authors to include several qualitative case studies comparing MedGuide-14B’s outputs with the corresponding clinical documentation and final diagnoses. In particular, presenting a small number of representative patients among the 4,226 newly identified HFpEF cases could illustrate which cues the model appears to leverage and why these may have been overlooked in routine care. Such case-level examples would make the findings more tangible and help elucidate why MedGuide-14B is able to identify additional cases beyond standard clinical practice.**

**Response:** We thank the Reviewer for this insightful and constructive suggestion. In response, we have added detailed qualitative, patient-level case examples to explicitly illustrate how MedGuide-14B integrates fragmented EHR information and identifies HFpEF cases that were not previously recognized in routine clinical care.

Specifically, we have introduced a new Supplementary Methods section (Section S3) presenting anonymized representative patient-level case vignettes, including (i) a clinically adjudicated HFpEF case as a gold-standard reference and (ii) a previously undiagnosed patient flagged by MedGuide-14B among the newly identified HFpEF cases. For each case, we provide the raw EHR inputs, the model’s structured diagnostic reasoning and outputs, explicit handling of missing diagnostic elements, and the final adjudicated interpretation.

These case-level examples demonstrate the specific clinical cues leveraged by the model—such as exertional dyspnea documented only in free-text notes, preserved LVEF with elevated filling pressures, and guideline-consistent but borderline H2FPEF or HFA-PEFF scores—and clarify how such signals are often distributed across disparate sections of the medical record, making them prone to being overlooked in routine clinical workflows.

In addition, we have expanded the Discussion to synthesize recurring qualitative patterns observed across MedGuide-identified HFpEF cases and to contextualize why large language model–based integration of heterogeneous EHR data may enable detection beyond standard clinical practice.

**Changes made in the manuscript:**

Supplementary Methods-Added Supplementary Section S4 (“Anonymized Patient-Level EHR Inputs and MediGuide-14B Outputs”) presenting representative qualitative case studies with raw EHR inputs, model outputs, missing-data handling, and final adjudicated diagnoses.

Discussion- Expanded the section on LLM-identified HFpEF and clinical interpretability to summarize recurring cues and explain why these are frequently overlooked in routine care (Pages 15,lines 363-373).

**4. Include a separate Literature Review section to includea more systematic and up-to-date overview of related work on large language models and other foundation models in cardiology and, more broadly, in clinical medicine.**

**Response:** Thank you for the suggestion. We have added a concise literature-summary section and a comparative table to place the current study in context with recent work on HFpEF identification in MAFLD and related cardiometabolic cohorts. The added table highlights differences in study populations, data modalities, methodological approaches, outcome definitions, and reported performance, thereby clarifying the specific contribution of our work.

**Changes made in the manuscript:**

Discussion-added a literature-context subsection on HFpEF under recognition in MAFLD and its relation to prior EHR- and ML-based studies (Page 14-15, Lines 352–372).

Table 4-added a comparative summary of representative studies from the past five years alongside the current study.( Page 27-28)

**5. For reproducibility, the manuscript should provide a clearer description of how MedGuide-14B was invoked (For example, the form of input and output, how it has assisted in the judgment of HFpEF) and how the experiments could be replicated by other groups.**

**Response:** We thank the Reviewer for emphasizing the importance of reproducibility. We have clarified how MedGuide-14B was invoked and how its outputs were used for HFpEF identification, including model inputs, standardized prompts, output formats, and classification criteria.

To support reproducibility, de-identified numerical data underlying all figures and tables, together with the standardized inference prompt and classification threshold, have been made publicly available in a Zenodo repository.

**Changes made in the manuscript:** Methods-Data processing and inference setup-added a clearer description of model inputs, standardized prompt structure, output format, and how probability scores were used to assist HFpEF judgment (Page 9–10,lines 220-227).

Data Availability Statement-added a public Zenodo repository link (https://doi.org/10.5281/zenodo.17970844) containing de-identified numerical data, standardized prompts, and threshold definitions to support reproducibility (Page 19,lines 476-478).

We sincerely thank the Editor and the Reviewers for their careful evaluation and constructive feedback. We have revised the manuscript accordingly and believe that the changes have substantially strengthened the clarity, rigor, and presentation of the study. We hope that the revised manuscript and the detailed point-by-point responses adequately address all concerns raised, and we would be grateful for any further suggestions.
